# A view on clinical genetics and genomics in Spain: of challenges and opportunities

**DOI:** 10.1002/mgg3.232

**Published:** 2016-07-18

**Authors:** Teresa Pàmpols, Feliciano J. Ramos, Pablo Lapunzina, Ignasi Gozalo‐Salellas, Luis A. Pérez‐Jurado, Aurora Pujol

**Affiliations:** ^1^ Division of Inborn Errors of MetabolismDepartment of Biochemistry and Molecular GeneticsHospital ClinicBarcelonaSpain; ^2^ Center for Biomedical Research on Rare Diseases CIBERER U737BarcelonaSpain; ^3^ Unit of Clinical GeneticsService of PediatricsUniversity Hospital “Lozano Blesa”ZaragozaSpain; ^4^ Functional GenomicsDepartment PediatricsUniversity of Zaragoza Medical SchoolZaragozaSpain; ^5^ Center for Biomedical Research on Rare Diseases CIBERER‐GCV02ZaragozaSpain; ^6^ Clinical Genetics UnitInstitute of Medical and Molecular Genetics (INGEMM)IdiPAZHospital Universitario La PazMadridSpain; ^7^ Center for Biomedical Research on Rare Diseases CIBERER U753MadridSpain; ^8^ Department of Romance LanguagesUniversity of Pennsylvania521 Williams Hall 255 S. 36th StreetPhiladelphiaPennsylvania19104; ^9^ Genetics UnitDepartment of Experimental and Health SciencesPompeu Fabra University (UPF)BarcelonaSpain; ^10^ Hospital del Mar Research Institute (IMIM)BarcelonaSpain; ^11^ Center for Biomedical Research on Rare Diseases CIBERER U735BarcelonaSpain; ^12^ Neurometabolic Diseases LaboratoryInstitute of NeuropathologyIDIBELLBarcelonaSpain; ^13^ Center for Biomedical Research on Rare Diseases CIBERER U759BarcelonaSpain; ^14^ Catalan Institution of Research and Advanced Studies (ICREA)BarcelonaSpain

## Abstract

A view on clinical genetics and genomics in Spain: of challenges and opportunities.

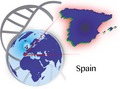

## Spain: The Great Transformation. A Geographical, Historical, Economic, and Social Overview

Historical and social perspectives, together with economic context, are essential to comprehend and appreciate the current status of any discipline. This is particularly true for fields relying on technological advances and with direct effects on society, such as genetics and genomics.

Spain or the Kingdom of Spain, the two denominations used to designate the country in international treaties, is a state of southwestern Europe that occupies most of the Iberian peninsula, together with Portugal, Gibraltar (an overseas territory of the United Kingdom), and Andorra, with archipelagos in the Mediterranean Sea (Balearic Islands), the Atlantic Ocean (Canary Islands), and the two cities of Ceuta and Melilla in continental North Africa. It is the fourth largest (504.645 km²) and sixth most populated country in Europe (48,146,134 inhabitants in 2015).

Politically, Spain is the youngest Western European democracy, exerted by a parliamentary government under a constitutional monarchy. The country has starred in two of the most extreme moments of Western European history in the twentieth century: the longest dictatorship in the modern era of the continent (1939–1975) and the greatest economic growth in the Eurozone since the creation of the European Union (1986–2008). In between these periods, during the so‐called *‘Transición Española,* 1 Spain began the most important sociopolitical progress of its modern history, starting with the death of the dictator Franco (1975).

The evolution of Spanish society must be analyzed from political and economic axes. There were three major periods in the past century of Spanish history: a first stage of suffering and scarring from the Civil War sank the country into deep poverty until the end of the autarchic Franco regime (1939–1958), a second phase of political and economic opening (1959–1977), and a third stage toward a fully democratic and parliamentary monarchy from the adoption of the Spanish Constitution (1978) until the present.

Franco's regime was established in 1939 after a 3‐year civil war that shook the entire world and anticipated the tragedy of the Second World War. This regime halted the social development of a country that in the first third of the twentieth century was implementing important structural changes to replace a failed political regime (*la Restauración* 2) by another more egalitarian regime (Republicanism). This political regression led to an economic and social downturn, fruit of the limitation of freedoms, and the obstruction of a rational social life.

From the end of the civil war to the sixties (1939–1959), Spain lived under an unusually long, politically isolated, autarchic economic model that caused severe economic hardship. However, despite the deep recession, Franco continued the Second Spanish Republic's attempt to create a Social Security system (1931) to ensure public healthcare and protect citizens’ rights to remunerated retirement. Although it was only a worker's privilege during the early dictatorship, Social Security became a right extended to all citizens several years later.

In 1959, Spain approved the national Stabilization Plan, which generated economic liberalization. Despite the dictatorial interventionism, this was the first period of remarkable socioeconomic progress in the twentieth century in Spain. Of note, most of Spain's main university hospitals were built from 1960 to 1975.

The next notable moment was the “Moncloa Agreements” (1982), where the various political parties in the Spanish transition agreed to stabilize the economy as a *sine qua non* condition for the modernization of the country. This paved the way for the third and definitive boost to the national economy when Spain joined the European Economic Community in 1986, which later became the European Union (BBC December 2015; IMF April 2016) and therefore the EU Common Market. The major contributors to economic growth were a decrease in oil prices in the mid‐1980s and a massive increase in foreign investment to expand the infrastructure and tourism of Spain (Gutiérrez‐Domènech May 2014; BBC 2015), and a *Reforma Laboral* or Labor Reform. Consumer demand was high in Spain, resulting in many imports from the EU, but exports remained limited, which led to a large trade deficit (Beltrán 2010).

With the worldwide contraction of the economy in 2008, the Spanish GDP suffered substantially. The construction bubble imploded, resulting in massive job losses and foreclosures. Unemployment peaked at 26.5% as a result of economic collapse, with youth unemployment at 56% in 2013 (IMF 2016). In 2010, the government announced several rounds of unpopular austerity measures, such as substantial tax increases and wage cuts to halt the growing deficit (Blanco 2010). Because of fears of default, the European Commission provided a 40‐billion Euro bailout package to restructure the weakest Spanish banks in 2012 (Reuters 2010). The public debt increased from 60.1% of the GDP in 2010 to 101% in 2015, which may have seriously handicapped Spain's future growth and development perspectives. The large underground economy added to weak, unsolved issues that hampered competition at the global scale.

Spain's current GDP of 1,615,074 million places it as the 16^th^ largest economy worldwide and the 5^th^ largest in the European Union (data from the International Monetary Fund, 2015). Despite slight signs of slow recovery in 2014 and 2015, it is not obvious whether the levels of public and private investment and social welfare will recover to their precrisis mark.

On a positive note, Spain does not suffer from a regression in the social rights of its citizens. The social rights of women, or minorities such as homosexuals or immigrants, are comparable with the most advanced countries worldwide. We hope that the current generation, the most educated the country ever had, contributes to preserve this precious legacy.

**Figure 1:**
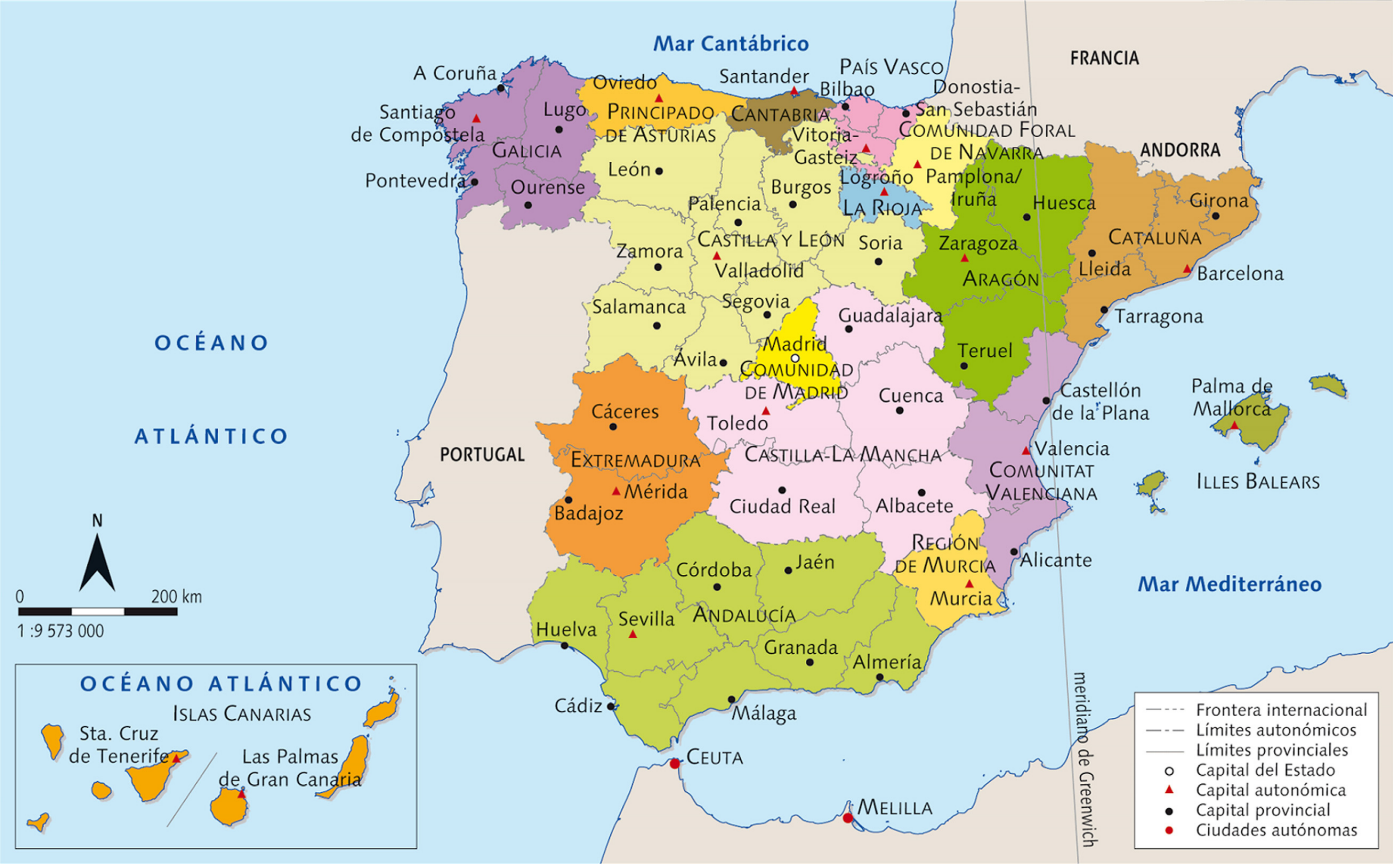
Territorial organization of Spain in Autonomous Communities or Regions (AC,* Comunidades Autónomas*).

Administratively, the Spanish state comprises 17 regions of autonomy, or Autonomous Communities/Regions (AC/R), and two autonomous cities in North Africa (Ceuta and Melilla). AC/R are the first‐level administrative divisions in the country created during the restoration of democracy at the end of 1979 (see Fig. 1). The AC/R have wide legislative and executive autonomy with their own parliaments, although only two AC/R, Basque country and Navarra, have full fiscal autonomy for historical reasons. The budget for education, research and development, and health services is allocated by the central government, and administered by each AC/R independently with authority to decide on the provision of health services in general, including genetic services and newborn screening. Nevertheless, there is an Interterritorial Council of the Spanish National Health System that harmonizes public health services and national strategies for several health problems, including the National Strategy for Rare Diseases (2015 World Health Report, Annual Report on the National Health System of Spain 2013, Informes, Estudios, e Investigación 2014, Ministerio de Sanidad Servicios Sociales e Igualdad; CIA The World Factbook; WHO‐Global Health Observatory).

Global healthcare indicators have steadily improved since 1970. The average life expectancy at birth, 79.3 years for men and 85.2 years for women (2013), is one of the highest in the world, and mortality rates have been decreasing to near the European Union averages. The neonatal mortality rate per 1000 live births is 2.8 (3.3), infant mortality is 8 per 1000 live births, and maternal mortality is 5/100,000 live births (for 413,000 live births year). The crude death rate per 1000 population is 9.04, and the top three causes of death in Spain are cardiovascular disease, cancer, and respiratory disease.

The SNS (*Sistema Nacional de Salud* – National Health System) care network has a total of 115,200 doctors and 165,000 nurses, mostly employed by hospitals. The SNS has a network of 453 hospitals, of which 325 are public. The physician density is 4.95/1000 people, and the total ratio of available beds is 3.0 for every 1000 inhabitants.

The SNS has 177 Reference Centers, Services, and Units (CSURS) in 42 healthcare centers to attend to certain pathologies or perform particularly complex procedures. Some of these CSURS are concerned with genetic diseases ( www.msssi.gob.es), although the applications for Genetics Clinics and Centers have not been opened yet.

The total expenditure of Spain's healthcare system is 95,670 million Euros, which represents 9.3% of the GDP. Of this share, 6.7% is funded by public resources and 2.6% comes from private resources. Regional governments (AC/R), which fund 91.7% of the expenditure, bear the greatest burden in the public funding of healthcare. Curative and rehabilitative care services comprise more than half of the total health expenditure (2015 World Health Report; Annual report on the National Health System of Spain 2013. Informes, Estudios, e Investigación 2014. Ministerio de Sanidad Servicios Sociales e Igualdad; CIA The World Factbook; WHO‐Global Health Observatory).

## Demographics, Population Diversity, and Genetics

The population median age is 41.4 years, with 77.4% of the population living in urban areas. The largest metropolitan areas are Madrid (6,052,247 inhabitants), Barcelona (5,030,679), Valencia (1,551, 585), and Seville (1,294,867).

Spain shows great cultural, religious, and linguistic diversity, partially because of immigration between 1995 and 2008 during the economic bonanza. Native Spaniards, ethnically composed of Mediterranean and Nordic types, constitute 88% of the current total population. The rest is composed by a community of around 1 million of Roma/gypsies and 4.6 million of foreign citizens, originating mainly from Latin America (39%), North Africa (16%), Eastern Europe (15%), and Sub‐Saharan Africa (4%) and also Middle Eastern countries as China, Philippines, Pakistan, India, and Bangladesh. The highest number of immigrant population concentrates in Catalonia, Madrid, Valencia, and Andalusia. (INE, Estadística del Padrón contínuo. www.ine.es).

Regarding official languages, Castillian/Spanish is the official national language. Catalan is official in Catalonia, the Balearic islands, and Valencia communities, where it is known as Valencian. Galician is official in Galicia, Basque is official in the Basque Country and in the Basque speaking area of Navarra, and Aranese is official in the northwest corner of Catalonia (Vall d'Aran) along with Catalan (<5000 speakers).

Roman Catholicism has long been the main religion, but it no longer has official status by law, and increasing secularism certifies the decline in the historical influence of the Catholic Church over the Spanish government. However, in all public schools, students must choose either an Ethics or Religion class, and Catholicism is the only religion officially taught. Today, Spain is religiously diverse, and there are other religious communities, such as Protestants, Jehovah's Witnesses, Mormons, Hindus, Buddhists, Sikhs, Muslims, and Judaists.

The current birth rate is 9.64 births per 1000 inhabitants and a growth rate of 0.89%. The mean maternal age is 32 years, and the total fertility rate is 1.49 children born/woman. Increasing parental age and low fertility rates suggest social changes, such as the incorporation of women into the workforce, insufficient measures supporting motherhood and family conciliation, gender inequality, and consequences of the economic crisis.

According to data recorded by the ECEMC (Estudio Colaborativo Español de Malformaciones Congénitas), the percentage of parents of foreign origin rose from 3.5% from 1980–1997 to 23.19% in 2008, which is expected to have a direct effect on the ethnic diversity of Spain in subsequent generations.

Notably, whereas the frequency of newborns with congenital defects is 1.4% for the native Spanish population, the global frequency is 1.52%, being significantly higher for other ethnic groups, most likely because of difficult sociosanitary conditions, different genetic substrates, and a higher number of consanguineous marriages (Bermejo Sánchez 2010). That study showed a linear decrease in the frequency of 15 congenital defects from 1980–1985 to 2008. More important decreases (expressed as the frequency of 10,000 newborns) occurred for anencephaly (4.65–0.28), spina bifida (4.73–0.66), and Down Syndrome (14.78–6.41). These results are related to different variables, including improved pregnancy care (i.e., folic acid supplementation), an increase in the healthcare culture of the population, and the voluntary termination of pregnancies after the detection of fetal abnormalities.

Currently, population registries of congenital defects in Spain are scarce (Salvador Peral et al. 1998), and it remains difficult to estimate frequencies of Mendelian disorders. A successful initiative in this regard was REDEMETH, created by the National Institute of Health Carlos III, Instituto de Salud Carlos III, ISCIII, which implemented a 3‐year survey (2003–2005) to collect 1675 cases from 158 different inborn errors of metabolism, quoting a cumulative incidence of 1 in 800 live births (Pampols 2010). Recently, a national registry for rare disease was created by the IIER (Institute for Research in Rare Diseases from the ISCIII) and integrated into the National Health System (Royal Decree 1091/2015 from 4 December).

In the past two decades, several studies have been published on differential particularities of several rare inherited diseases in the Spanish population: (1) the relatively high prevalence of mild phenylketonuria (PKU) phenotypes in Spain compared to Northern European populations (Desviat et al. 1999); (2) the large mutation heterogeneity causing cystic fibrosis (CF), for which the ΔF 508 mutation accounts for only 48% of CF alleles compared to 70% in Central and Northern European Caucasians (Casals et al., 1993); (3) the higher prevalence of alleles causing autosomal recessive (35delG in conexin 26) or mitochondrial inherited deafness (A1555G in the 12S rRNA) in the Spanish population compared with Central and Northern European countries (Estivill et al. 1998; Gasparini et al. 2000); and (4) the high risk of MCADD, Canavan, and GM1 gangliosidosis in the Roma/gypsies population (Martínez et al. 1998; Santamaría et al. 2006) together with the observation of many other recessive disorders in this ethnic group due to high inbreeding (López‐Meseguer et al. 2015; Tenorio et al. 2015).

The use of next generation sequencing (NGS) technologies has recently confirmed that the allelic frequencies of variants conferring susceptibility to complex diseases, such as neuropsychiatric, neurodegenerative, cancer, or type 2 diabetes, are not different from other European populations (Dopazo et al. 2016). However, the concept of relative homogeneity within European populations has been questioned by genome‐wide association studies (GWAS), which report a direct correlation between genetic and geographical distances (Novembre et al. 2008). Recent research of Dopazo based on the whole exome sequencing (WES) of 267 healthy Spanish individuals indicated that the level of variation in local populations, even in the absence of geographical barriers, is higher than expected from previous studies based on polymorphisms (Bustamante‐Aragonés et al. 2011). A large portion of this variability corresponds to private variants that confer susceptibility or directly cause disease in the Spanish population for many Mendelian and rare diseases, such as hereditary cardiomyopathies, Marfan syndrome, and degenerative retinal dystrophies (Dopazo et al. 2016). A recent study aimed to identify individuals carrying recessive mutations among gamete donors for reproductive medicine has also contributed to establishing carrier frequencies for the most common genetic disorders in Spain (Abulí et al. 2016). Thus, allele frequencies indicate significant local effects on disease‐causing variants that must be accounted for when vetting candidate variants of Spanish or Latin‐American populations (Dopazo et al. 2016). This result emphasizes the need for population‐specific catalogues of genetic variation when attempting diagnoses and phenotype–genotype correlations. We anticipate that these approaches will deliver essential information for improving genetic diagnosis and disease management.

### Prenatal diagnosis and prenatal screening

In Spain, the genetic analysis of prenatal samples has followed a simple rule from the beginning: it had to be developed in genetic services already offering postnatal diagnosis, provided they had acquired technological expertise and felt comfortable with the interpretation of fetal samples. The first prenatal cytogenetic diagnostics in cultured amniocytes were performed in the late 1970s; in 1982, the first biochemical genetic diagnosis was successful. However, when an affected fetus was identified, pregnancy termination could not ensue because abortion was considered a crime until 1985.

In that year, article 417 *bis* of the penal code was modified, establishing that abortion would not be penalized in three situations: (1) if there is risk to the life or health of the mother; (2) if the fetus is expected to be born with severe physical or psychic disorders; and (3) rape (Ley orgánica 9/1985 de 5 de julio de reforma del artículo 417 *bis* del Código Penal). In 1986, the Ministerial order of July 16th provided statistical and epidemiological data on the voluntary interruption of pregnancy. A Royal Decree from November 21st of the same year provided data on the accreditation of centers for the legal practice of voluntary interruption of pregnancy. Then, the General Direction of Public Health of the Ministry of Health developed a program for prenatal diagnosis that was included in the National Plan for the prevention of intellectual disability (*Plan Nacional de Prevención de la Subnormalidad*), the same plan that provided for newborn screening (see above). At this time, 13/17 of the AC had between 1 and 8 centers of prenatal diagnosis, in which 13,240 karyotypes in cultured amniocytes, 1751 studies in amniotic fluid, 117 in chorion villi, and 6030 consultations of genetic counseling were performed (*Programa de diagnóstico prenatal. Estudio de los recursos existentes en el área de genética. Dirección General de Planificación Sanitaria. Ministerio de Sanidad y Consumo, 1986*).

In 1987, the Spanish Society for Prenatal Diagnosis (AEDP) ( www.aedprenatal.com) was created, and the first public health programs for the screening of congenital defects emerged in the 1990s. This included a triple screening (biochemical + maternal age + echography, followed by karyotype for positive cases) for the detection of neural tube defects and chromosomal disorders.

In 1997, a survey generated by AEDP analyzed a geographic area with 342,619 births (total births in Spain 350,000) and showed: (1) important territorial inequalities, in which a geographic area with 117,000 births remained uncovered by any Public Health Program, and (2) eight AC/R did not assume perfectly legal abortions because they conflicted with conscience objection, also recognized by law. Abortion was selected in 528 cases with severe congenital defects, which explained only 1.06% of the 49,758 lawful abortions during the identical period. In two cases, rape occurred, and the physical or psychological health of the mother (certified by two independent psychiatrists) was adduced as the reason for termination in most cases, a good indicator of complex underlying social realities (Farrán and Pampols 2000).

Important changes occurred in 2010. The Organic Law 2/2010 recognized the right of women to voluntarily interrupt undesired pregnancies under some conditions: (1) if it occurs before 14 weeks of pregnancy; (2) the woman must be previously informed of rights, benefits, and public help in support of motherhood; and (3) a period of at least 3 days must be granted to reconsider and reflect on information and intervention. The detailed evaluation of the enforcement of this law shows significant data, as the total number of voluntary abortions decreased from 113,031 to 94,796 in 2010. Undesired pregnancies accounted for 88.90%, severe risk for the life or health of the pregnant woman was up to 7.15%, the risk of severe abnormalities in the fetus was 3.61%, fetal abnormalities incompatible with life or extremely severe or untreatable diseases was 0.32%, and other reasons accounted for 0.01% (*Interrupción voluntaria del embarazo. Datos definitivos correspondientes al año 2014. Ministerio de Sanidad, Seguridad Social e Igualdad*). Of note, the overall frequency of newborns with congenital defects dropped from 2.22% in 1980–1985 to 1.03% in 2010, mostly as a result of voluntary termination. However, certain defects show a secular increase arising from social causes, such as the progressive increase in parental age. Ethnic diversification resulting from immigration is also having an effect (Bermejo Sánchez 2010) (*Estudio Colaborativo Español de Malformaciones Congénitas (ECEMC) www.Fundacion1000.es*).

Prenatal diagnosis using cytogenetics, molecular genetics, and biochemical genetics is regularly performed in the public health system. New technologies, such as chromosomal microarrays (Armengol et al. 2012) and the study of fetal DNA in maternal plasma, are also being implemented (Bustamante‐Aragonés et al. 2006). The use of these and other emerging technologies challenges the current prenatal screening strategies (Del Campo Casanelles et al. 2015; Plaja Rustein et al. 2015) and must be considered with the analysis of sociohealth conditions when designing prevention plans for these diseases.

## Newborn Screening

Blood‐spot newborn screening (NBS) in Spain dates from 1968 to 1969, at which time Pr. Federico Mayor Zaragoza (who would later be President of UNESCO) at Granada University and Dr. Joan Sabater at the Institut de Bioquímica Clínica in Barcelona began offering NBS for phenylketonuria and other amino acid disorders using existing paper chromatography and thin‐layer chromatography methods. Other centers soon followed this initiative, but the prevention of intellectual disability at the national level was adopted in 1976 with the creation of the *Real Patronato de Educación Especial* then *Real Patronato de Educación y Atencion a Deficientes* under the presidency of Her Majesty Queen Sofía, who nominated Dr. Federico Mayor Zaragoza to head the National Program *Plan Nacional de Prevención de la Subnormalidad*. This plan established objectives in three areas: Metabolic/Genetic, Nutritional/Pediatric, and Perinatology. The first area included newborn screening and the diagnosis of chromosomal abnormalities. After several organizational changes, the *Plan Nacional de Prevención de la Subnormalidad* was assumed and financed by the Ministry of Health; by 1980, there were 10 NBS centers covering 25% of Spanish newborns and several cytogenetic laboratories in public hospitals.

Spain's administrative structure allowed AC/R to independently decide what diseases to include in their newborn screening programs. Until 2000, all autonomous governments included congenital hypothyroidism (CH) and PKU. In some AC/R, the program also included, although not uniformly, congenital adrenal hyperplasia (CAH), galactosemia (Gal), cystic fibrosis (CF), biotinidase deficiency (BD), and sickle cell disease (SCD).

Large changes arrived in 2001 when Galicia expanded its program with tandem mass spectrometry (MS/MS), which was soon followed by Murcia and Andalusia. Other AC/R delayed the decision to expand programs with MS/MS for different reasons, such as the lack of cost effectiveness for such low prevalence diseases in small screening centers, limited knowledge of medicine‐based evidence of benefits, and low predictive positive value for some diseases.

The situation rapidly evolved, and in 2012, as many as nine diagnostic centers screened between 2 and 10 diseases, seven screened between 19 and 28, and three screened between 32 and 47 (*Informe del grupo de expertos sobre concreción de cartera común de servicios para cribado neonatal. Ministerio de Sanidad Servicios Sociales e Igualdad. 2012*).

Despite disparities on the information received by the parents, the type of consent, and the magnitude and volume of processed samples per laboratory or methodology used, important coincidences were evident: the NBS was developed as a Public Health Program in all AC/R, being universally and fairly offered to the whole target population; despite not being mandatory, the compliance rate was nearly 100%. The integrated programs included clinical diagnosis, treatment, and follow up, and they were all deeply concerned with quality assurance issues, participating in national and international external quality controls and were progressively certified under the norm UNE‐EN ISO 15.189.

In 2012, the Interterritorial Council of the Spanish National Health System updated the Common Portfolio of Services for the presymptomatic detection of diseases in the screening population constituted by two working groups: (1) cancer screening and (2) newborn screening.

The working group for NBS included professionals representing AC/R; scientific societies; the Spanish Network of Technology Evaluation Agencies; and the Ministry of Health, Social Services, and Equality. The first resolution was the adoption of criteria to include a disease in a program: 18 criteria according to a frame document (*Documento marco sobre cribado poblacional. Ponencia de cribado poblacional de la comisión de salud pública. Ministerio de Sanidad Servicios Sociales e Igualdad. 2010*). The SSI/2065/2014 law from October 13^th^, 2012 established that screening for seven diseases must be offered to the target population in all AC/R: CH, PKU, MCADD (Medium Chain Acyl‐CoA Dehydrogenase Deficiency), CF, SCD, LCHADD (Long‐chain L‐3 Hydroxyacyl‐CoA Dehydrogenase Deficiency), and GA type I. Among inherited metabolic disorders detected by newborn screening, the most frequent are PKU (1: 18,280) and MCADD (1: 20,158), and the most rare is MADD (Multiple Acyl‐CoA‐Dehydrogenase Deficiency (1: 516,793)). Hence, the incidence of disease was not accounted for as an inclusion criterion in NBS policy but rather the availability of efficacious treatment and a robust biomarker correlated with the phenotype. Currently, other diseases are being evaluated for future inclusion in the programs.

The past few years have been characterized by an increasing awareness of ethical, legal, and social aspects, which have prompted the publication of documents with ethical recommendations aiming to promote public debate in the hopes to contribute to responsible guidance (Abascal et al. 2009; Pampols Ros et al. 2010) and the enforcement of Law 14/2007 of Biomedical Investigation (LBI). This law aims to encourage biomedical research while ensuring the highest possible level of health and ethical guarantees to protect society. Therefore, it regulates genetic testing and genetic screening in biomedical research and its healthcare applications. Other dispositions establish that: (1) the program must be evaluated by an ethical committee; (2) participation requires written consent, but consent may be expressed verbally in exceptional occasions if granted by the ethics committee; (3) prior information must be written and shall refer to the voluntary nature of participation; (4) the quality of screening tests, diagnostic tests, and treatment and follow‐up must be ensured with checkpoints to follow‐up on entire programs; (5) the importance of psychosocial aspects of the program is stressed; (6) the program must be integrated into the health system; and (7) the same rules established for genetic testing apply to the screening tests, which includes the provision of genetic counseling.

The LBI also regulates the use of human biological samples for biomedical research. The use of residual blood‐spot samples and associated data for research purposes and their indefinite storage require explicit consent and an ethical review board. The more frequent situation is the short storage of residual blood spots, typically 1–3 years, to permit diagnosis, retesting to confirm results, post mortem diagnosis, and laboratory audit and quality control, which do not require explicit consent.

In conclusion, it is currently mandatory by law in Spain to offer screening for the seven diseases included in the Common Portfolio of Services, but participation is voluntary. NBS is typically conducted without explicit consent because it is considered to be in the best interest of a child's health and part of routine pediatric practice. The more common situation is an “opt‐out” system, whereby screening is applied unless parents refuse. According to LBI Article 4. *Informed consent and the right to information*, the heterozygous state of a recessive disease can be communicated if the “right not to know” is respected and genetic counseling is provided. This implies the anticipation of this possible result in the information provided to the parents and the acceptance of the possibility to refuse this information. According to this article, it could also be inferred that parents have the right to be informed of incidental results regarding off‐target diseases when they are clinically actionable. Information on Spanish screening centers, including statistics and disease incidence, from the beginning of the programs can be located at the Spanish society for newborn screening (AECNE) website ( www.aecne.es).

## Assisted Reproductive Technologies and Human Genetics

The elaboration of reproductive decisions is a subject in which the social perspective is deeply rooted in values and beliefs, and the interface between assisted reproductive technologies and genetics raises important technical, social, ethical, and legal issues (Soini et al. 2006).

In Spain, assisted reproduction techniques (ART) are socially well accepted, legally authorized, and included within the services offered by the public health system, although the bulk of activity is performed in private centers, several with international prestige. According to a survey by the Spanish Association of Human Genetics (AEGH), only 5/13 responding centers were public, and preimplantation genetic diagnosis (PGD) had been performed for 99 different diseases (*Informe Situación del diagnóstico preimplantacional, Comisión para el diagnóstico preimplantacional, Julio 2012,* www.aegh.org). Instituto Dexeus, a private center, housed the first baby born after in vitro fecundation, with Dr. Anna Veiga, currently the Chairman of the European Society for Human Reproduction and Embriology (ESHRE). The Register of Centers and Services in Spain, both public and private, can be located at www.sefertilidad.net.

Spain is the European country with the most cases of assisted reproduction, with 17,011 cycles in 2013 (Registry of the Spanish Society of fertility, www.sefertilidad.net). Spain is one of the first countries with a law on ART (Law 35/1988 from November 22 on assisted reproduction techniques). This law and two other posterior laws have been derogated by Law 24/2006 from 28 May with successive modifications and a consolidated text in 2015 (*Ley 14/2006, de 26 de mayo sobre técnicas de reproducción humana asistida, Texto consolidado, Ultima modificación 14 de julio de 2015*).

The law considers that the use of ART and PGD for embryonic selection applies in the following cases: (1) the avoidance of severe diseases with early presentation and without treatment; (2) the avoidance of diseases presenting at 30–50 years (Huntington Disease, cancers with mild or high penetrance); and (3) for therapeutic purposes (the selection of an offspring‐compatible donor for a previously sick child). The two last applications require case‐by‐case authorization based on previous reports from the National Commission of Human Assisted Reproduction ( www.cnrha.msssi.gob.es). Sperm and oocyte donors should remain anonymous; this requirement can be waived under exceptional circumstances, although this is currently a subject of ethical and legal debate. Donation must be altruistic, but economical compensation is allowed to cover alimentary expenses, travel fees, or loss of working hours, mainly for oocytes donors. The fate of surplus embryos can be decided by the woman or couple, including their use for research purposes. Preimplantation diagnosis for sex selection is allowed to avoid X‐linked diseases but not for social or family balancing reasons.

There are other legal dispositions relevant for ART, including Law 16/2003 of Cohesion and Quality of National Health Service. Law 14/2007 from 13 July of Biomedical research (LIB) provides legislation on donation and the use of embryos, fetuses, and cells and tissues from human embryonic origin, and the Royal Decree Law 9/2014 from 4 July establishes norms of quality and safety for the donation, obtainment, evaluation, processing, preservation, storage, and distribution of human cells and tissues and approves the norms for the coordination and functioning of its use in humans. Of note, Spain is one of the few European countries (together with Greece, Cyprus, and Poland) in which oocyte donation for women with advanced maternal age and low ovarian reserve is allowed, although women must be warned of the risks arising in pregnancies at clinically advanced age. Concerning the option for avoiding maternally inherited mutations in mitochondrial DNA, the so‐called “three parent embryo” is not currently allowed, as the techniques of nuclear transfer with reproductive finalities is not permitted; thus, oocyte donor programs remain the only current option.

## Genetic Services and the New Clinical Genetics Specialty

The first geneticists in Spain were physicians who introduced Medical Genetics and Cytogenetics to the health system at the end of the 1960s. Other professionals, such as biologists and pharmacists, were soon incorporated into clinical genetic laboratories. However, formal training in the different clinical genetics figures has been badly delayed in the country; as a result many professionals engage in reputed training programs abroad.

The Spanish Society for the Study of Human Genetics, currently the AEGH, was created in 1974. At that time, the society included 66 physicians, 12 biologists, and three pharmacists. Only 3 years later, there were 128 biologists, 87 physicians, seven pharmacists, and one chemist. In 2002, there were 200 biologists, 97 physicians, 34 pharmacists, and 39 professionals from several other fields, such as chemistry, biochemistry, and biotechnology.

Genetics training was part of pediatrics in medical school, and laboratory genetics was studied partially in medicine but also in the faculties of pharmacy, with a long tradition in clinical analysis and biology.

Of relevance, the Spanish Society of Clinical Genetics and Dysmorphology (Sociedad Española de Genética Clínica y Dismorfología –SEGCD‐) is integrated by pediatricians (mostly) and other specialists or health professionals who work in the clinical genetics units of Spain's major hospitals. The society was founded in 1978 as a Section of the Spanish Association of Pediatrics (AEP) and in 2002 was credited as an independent Society, working very closely with the AEGH, sharing knowledge with the laboratory geneticists and genetic counselors in order to deliver the standardized excellent care in genetic diseases to the patients and their families.

The claim for accredited health professionals in the genetics specialties in Spain is as old as AEGH, with the first request to health authorities made by its first president. Fifteen years later in 1989, AEGH promoted a specific commission for the specialty, and the National Commission for Medical Specialties approved the specialty of genetics. Nevertheless, strong conflicts arose between physicians and nonphysician professionals, and the specialty of genetics was paralyzed.

In 1996, the European Society for Human Genetics launched a European project directed by Professor Harris, the Concerted Action on Genetic Services in Europe (CAGSE), in which AEGH actively participated. According to AEGH, there were 40 centers offering cytogenetic services and 25 offering molecular genetics services. Biochemical genetics services were not recorded, but there were at least six laboratories offering diagnosis of inborn errors of metabolism. Seventy‐three percent of laboratories were in the public health system, but private centers began to appear. Nevertheless, the CAGSE project showed that the clinical genetics situation in Spain was one of the worst in Europe due partially to the lack of organization in the health system and the absence of specific academic education programs (*Genetic Services in Europe, A comparative study of 31 countries by the Concerted Action on Genetic Services in Europe, Editor and Project Leader Rodney Harris. 1997*; Ramos‐Arroyo et al. 1997).

CAGSE recommended the official recognition of medical genetics as a medical specialty in Europe, and in 2001, the European Society for Human Genetics (ESHG) promoted its formal and international recognition. At that time, the Spanish health system contained important organizational gaps in genetics, but within 5 years, the number of centers with molecular genetics services doubled (52 centers/laboratories offering diagnosis for 214 diseases) (Rueda and Briones 2002).

In 2002, the President of AEGH again requested that the Ministry of Health formally recognize the specialty but was denied by the National Council for Medical Specialties. AEGH approved the creation of a new commission for the specialty coordinated by its President, and integrated by five professionals with different academic backgrounds, including medicine, pharmacy, and biology; multiple years of work in the public health system; experts in Medical Genetics and Genetic Counseling, Cytogenetics, Molecular Genetics, and Biochemical Genetics; and on population screening. The AEGH Commission always offered well‐prepared documents, including a program proposal for specialty training, and was always present in all concerned forums, progressing slowly (*Propuesta razonada para justificar la oportunidad de crear la especialidad de genética en el actual Sistema Sanitario del estado español, 2005*;* Propuesta de programa de formación de especialistas en genética clínica, 2005*).

Between 2007 and 2010, important events occurred in Spain that stressed the effect of human genetics on healthcare and the need for qualified genetic services: enforcement of the above‐mentioned Law 14/2007 of Biomedical Investigation (LBI) that among other important functions, regulated genetic testing and genetic screening in biomedical research and healthcare applications; the launch of the National Strategy for Rare Diseases in the National Health System, indicating that 3,000,000 Spanish citizens could be affected by a rare genetic disease; Spain became a partner in EUROPLAN, the European Project for Rare Diseases National Plans Development; and CIBERER was born (see below).

The importance of LBI for genetic testing must be stressed: it provides criteria for genetic analysis, including pertinence, quality, equity, and accessibility; it specifies that written consent is necessary to undergo genetic analysis; it recognizes the patient's right to information and the “right not to know,” outlining the duty of confidentiality and the right to the protection of genetic data; it warns against the possibility of unexpected results and the decision of receiving or not its communication; it cautions that the information obtained may have implications for family members and when it is appropriate to convey information to them; and it states the compromise to provide genetic counseling (Pampols et al. 2013).

In Europe, members of Eurogentest Unit 6 and ESHG Education Committee developed core competencies to support the preparation of geneticists, and the Committee of Ministers to Member States adopted recommendation CM/Rec (2010)11 on the effect of genetics on the organization of health services and training of health professionals. Of the 27 European Union members, Spain was still the only one without a medical specialization in genetics, educational courses from an accredited institution for genetic professionals, or a common organization for genetic services in the different AC/R (Skirton et al. 2010; Tejada 2010).

At this time, the Ministry of Health is preparing important changes to the training program for medical specialties. Many programs will have a 2‐year common core for those specialties sharing affinities before differentiation and preparation in specific competencies. This reform is a priority, and new specialties must wait its approval (*Proyecto de Real Decreto por el que se regula la incorporación de criterios de troncalidad en la formación de determinadas especialidades en Ciencias de la Salud, La re especialización troncal y las áreas de capacitación específica, Ministerio de Sanidad, Política Social e Igualdad, Madrid, 2010*). AEGH adapted its proposal to this scheme (*Propuesta razonada para justificar la oportunidad de crear la especialidad de genética en el actual sistema sanitario del estado español e incorporarla al sistema de troncalidad. 2007,* www.aegh.org *, Comisión de la Especialidad*).

In 2011, the amending annexes II and V to directive 2005/36/EC of the European Parliament and of the Council on the Recognition of Professional Qualifications included a Clinical Genetics specialty and represented crucial support (Commission Regulation (EU) No 2013/2011 of March 2011 amending annexes II and V to Directive 2005/36/EC of the European Parliament and of the Council on the Recognition of Professional Qualifications).

In July 2011, a Project of Royal Decree recognized the Clinical Genetics specialty (*Proyecto de Real Decreto por el que se crean nuevos títulos de especialista y se actualiza el sistema formativo de determinadas especialidades en ciencias de la salud. Ministerio de Sanidad, Política Social e Igualdad. Madrid, 2011)*.

In 2012, ESHG recognized Clinical Laboratory Genetics as an EU‐recognized specialist profession, but in Spain, the Clinical Genetics specialty was unique and assigned under the umbrella of the professional stem group called Laboratory and Clinical Diagnosis.

Finally, in August 2014, a Royal Decree was enforced with the creation of two new specialties: Child Psychiatry and Clinical Genetics (*Real Decreto 639/2014 de Troncalidad y Creación de Nuevas Especialidades del Sistema Nacional de Salud en España*). A National Commission for the Specialty of Clinical Genetics was created to advise the Ministry of Health. Its nine members, nominated by different agencies such as Universities, Professional Colleges, or Scientific Societies, will have, among others, two main initial tasks: (1) the preparation of the training program for the new residents, and (2) the selection of the criteria for accreditation of training centers and resident's tutors.

In parallel, the *European Board of Medical Genetics* (EBMG) is currently working on harmonization, modernization, and recognition of the specialty at the European Union level, following the scheme of three professional areas: Clinical Genetics, Laboratory Genetics, and Genetic Counseling. Spain aims to contribute to this concerted action for the creation of all three Specialties in Europe.

While waiting for the first generation of Spanish Clinical Genetics specialists, some AC/R governments have launched plans for genetics (*Plan de Genética de Andalucia, Plan para el desarrollo de la genética en la Comunidad Autónoma del País Vasco, Instrucció 06/2015 Servei Català de la Salud*). Many academic hospitals in the healthcare system are organizing new integral and integrated genetic services with links to research that promote continuous technological development that is translated to healthcare. Somatic cell genetics and pharmacogenetics are progressing fields, and a common portfolio of genetic services is being developed by the Ministry of Health (*Orden SSI/2065/2014 de 13 de Octubre*). Actions on rare disease and the enforcement of Law 14/2007, which calls for the provision of genetic counseling once the results of the analysis are obtained, have favored education on this aspect, including the European Master of genetic counseling from the University “Pompeu i Fabra” and the creation of SEAGEN, the Spanish Society for Genetic Counselors ( www.seagen.es).

Similar to clinical genetics and despite the increasing demand of care for patients affected by rare diseases and cancer, the profession of genetic counselor has not been yet formally recognized in Spain. In general, genetic counseling is mainly provided by professionals working in the established Genetic Services and familial cancer units along with other medical specialists at the hospitals to date. Thus, a shortage of trained health professionals in the field was evident, particularly in light of the rapid development of genetic and genomic testing.

A specific educational program to train genetic counselors was established during the academic year 2007–2008 in the life sciences school of the Universitat Pompeu Fabra in Barcelona, under the direction and coordination of medical geneticists trained abroad. This program, which remains the only training program in Spain, leads to a Master Degree specialized in Genetic Counseling. This highly competitive 2‐year full‐time program provides a high level of knowledge in both scientific and medical fields, along with the required competences dealing with the psychological, social, and ethical aspects of genetic counseling. The Master Program has been accredited by both the Agency for Quality of Universities from the National Education Ministry and by the specific section of the European Board of Medical Genetics (EBMG, https://www.eshg.org/408.0.html). Currently, it is one of the only six programs in Europe that obtained the certification for training genetic counselors by the EBMG, being also part of the Transnational Alliance for Genetic Counseling (TAGC, http://tagc.med.sc.edu/). As a consequence, although the profession is not yet supported by a legal framework in Spain, more than 40 genetic counselors have graduated from the accredited educational program and are progressively being integrated as full members on the multidisciplinary teams of many public and private Centers dealing with genetic diseases and conditions. In addition, the Spanish Society for Genetic Counselors was created in 2011 (SEAGEN, www.seagen.es) and is further contributing to the recognition of the profession. Of note, Spain is currently, along with France, the European country with more genetic counselors accredited by the EBMG. Spanish genetic counselors are also involved in the evolution of educational and professional standards in Europe and are working actively for the certification of genetic counselors as members of the EBMG.

With the advent of Genomic Medicine, Spain is well prepared for genomic analysis, with state‐of‐the‐art public centers, such as the Spanish National Genotyping Center ( www.vsc.es/cenag), Center Nacional d'Anàlisi Genòmic ( www.cnag.cat), and the Genomics and Bioinformatics Platform of Andalusia (GBPA). The challenge remains to deliver genomic medicine promises to the public health system and to ensure high‐quality genetic diagnostic and management as recommended by law (*Ley 16/2003 de 28 de mayo de Cohesión y Calidad del Sistema Nacional de Salud)*. This will require the concerted action of political and social authorities to include WES and WGS in the portfolio of tests reimbursed by the national health system. Moreover, current tools for cooperation among Autonomous Regions without administrative barriers for the quality, equity, and accessibility of all Spanish citizens to a standard‐of‐care must be improved (*Ley 16/2003 de 28 de mayo de Cohesión y Calidad del Sistema Nacional de Salud)*.

## Research on Clinical Genetics and Genomics: The CIBERER

To better understand the current challenges faced by the scientific and medical community in one of Europe's youngest democracies, it is useful to gain perspective by providing a historical note on the fate of Spanish science in the past 100 years. In the words of Emilio Muñoz, former President of the prestigious *Consejo Superior de Investigaciones Científicas,* CSIC (and Emeritus Research Professor at the Institute of Philosophy of CSIC): “There has never been a Spanish golden age of science and technology. We can only talk about a Spanish silver age between 1880 and 1936. In this period, Spain was going through an era of regeneration where education, science, and technology were promoted by organizations, such as the Free Education Institution (*Institución Libre de Enseñanza*) and the Board for advanced studies and scientific research (*Junta para Ampliación de Estudios e Investigaciones Científicas* – JAE). During this period of change, the figure of Santiago Ramón y Cajal became a pioneer of science policy in Spain. To date, he remains the only Spanish scientist awarded with a Nobel Prize for studies in Physiology and Medicine performed within Spain.”

The Spanish Civil War (1936–1939) led to the dissolution of JAE and the exile of many in the academic and scientific community. As early as 1939, a commission of technocrats created the National Scientific Research Council (*Consejo Superior de Investigaciones Científicas, CSIC*) in the first significant strategic effort to push modern Spanish science and technology. This organization was under the control of Opus Dei, a conservative Catholic institution, which defended the literalism of the Bible, thus considering Darwinism unacceptable. As an example, in the final years of Franco, religious censors prohibited the great naturalist and science broadcaster Félix Rodríguez de la Fuente, also known abroad as the Spanish Jacques Cousteau, from using the phrase “the sea, the cradle of life” on public television (Editorial 2013). Also in the terminal years of the regime, an attempt to establish the basis for research and development arrived with the creation of the Technological Institute of Postgraduates (ITP). This project sought to copy the private funding model for scientific research of the Massachusetts Institute of Technology (MIT). Remarkably, the idea did not get enough support by the first democratic government of Adolfo Suárez. Finally, the socialist government ended the project during the 1980s.

After Franco's death in 1975 and several years of transition to democracy filled with political tension after a frustrated military coup in 1981, some individual initiatives crystallized upon the arrival of the Socialist Party in government in 1982. Spain finally joined the European Community in 1986 and started to build a research infrastructure in alignment with those of more advanced European countries. In particular, two granting agencies were created, one dedicated to funding fundamental science (MINECO, depending on the Ministry of Science and Innovation and mirroring the French CNRS), and the second dedicated to funding translational and clinical research (*Fondo de Investigación Sanitaria from the Instituto de Salud Carlos III*, depending on the Ministry of Health and Social Policy and inspired by the French INSERM). In recent decades, the CSIC has regained the JAE spirit of international exchange and freedom of thought, reaching a high scientific level. Technology transfer and innovation in the industrial sector lagged behind until now, with the economic power in the country traditionally reluctant to significant investments in R + D + i. However, a positive trend of increased governmental investment was noticeable in the first years of the new millennium, until it plateaued in 2008 with 1.9% of the governmental budget allocated to research. Such efforts, together with direct funding from the European Commission, led to the building of prestigious research institutes and infrastructures (CNIO, PRBB; Barcelona Science Park, CABIMER, ICFO among others), the consolidation of national scientists, and the hiring of internationally recognized scientists through highly competitive programs, such as ICREA or Ikerbasque, to lead a new era and boost scientific production in Spain to positions in accordance with its demographic and economic weight. This policy was productive until the unprecedented economic crash of 2008 resulted in a sudden, draconian cut of 40% of research funds, which is sustained today. Structural damage to all research bodies ensued, which many believe is nearly irreversible (Moro‐Martin 2012, 2014), along with other austerity measures that affected even the crown jewel, the public hospitals and diagnostic services belonging to the National Health System, who depend largely on funds managed by the AC/R. Many large hospitals across the country had to close entire floors and operation rooms and saw the waiting list for essential interventions increase dangerously. Currently, the Spanish government has not recovered from the crisis and dedicates a meager 1.3% of its budget to research (2013). This is far from the 2% investment of traditionally wealthier countries, such as the USA, Switzerland, Germany, or Northern European countries; even farther from the desirable goal of 3% proposed at Lisbon's Treaty in 2009; and below the average of 1.4% in the Eurozone (see Eurostat data for 2014).

This confirms that the importance of science, technology, and innovation as drivers of social and economic progress is not fully recognized by policy makers, differing from more advanced countries worldwide. Critical voices noted that the unprecedented growth from 1995 to 2007, largely due to an increase in consumer needs and a massive housing and construction bubble, did not lead to investments in long‐term policies to favor research, development, or the industrial sector but rather to further expand the solid tourism infrastructures and tertiary sector, thus resulting in a large quantity of low‐quality, volatile jobs. Furthermore, the minimum requirement of an independent commission of scientific experts to assist Congress on scientific, technologic, and innovation policies remains absent. The Ministry of Science has also recently disappeared, being currently reduced to a secondary branch of the Ministry of Economy. Thus, beyond a lack of funding, the necessary structures required to ensure a stable, long‐term framework to make science and innovation the central axes of a more robust and sustainable model of economic growth and social welfare are simply not yet in place.

**Figure 2:**
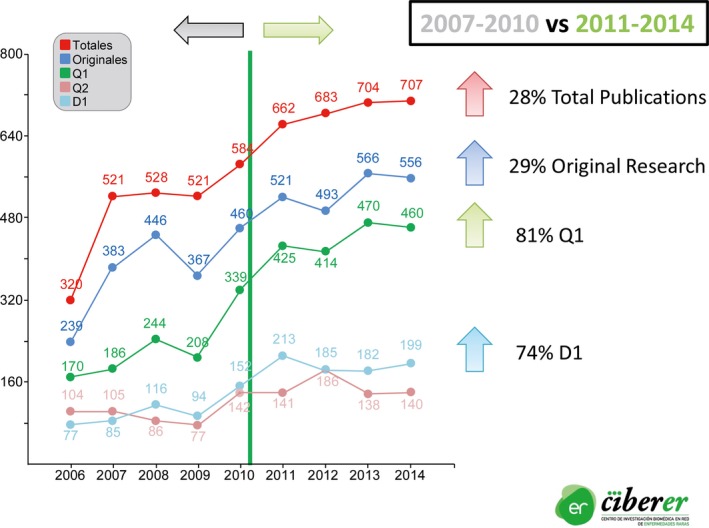
Scientific publications of CIBERER (2006–2014). Important increases in the number and impact factor publications of the research groups comprising CIBERER since its inception, underscoring the value of collaboration and structure. D1: first decile, Q1: first quartile, Q2: second quartile.

In this grim scenario, a light appeared. A public consortium called CIBERER (Center for Network Research on Rare Diseases) emerged in 2006 under the auspices of the Spanish National Institute of Health Carlos III (ISCIII), right before the global economic crash. This innovative research structure is a member of a broader umbrella comprising another 11 consortia dedicated to the main priorities in the National Health System, such as Obesity and Nutrition, Diabetes and Metabolic Disease, Hepatic and Digestive Disease, Respiratory Diseases, Epidemiology and Preventive Medicine, Mental Health, Neurodegenerative Diseases, and Bioengineering, Nanomedicine, Cancer, Cardiovascular Diseases and Aging. The CIBERER provides a safe haven to the best established and also for emerging research groups working on rare diseases. Today, it is comprised of 62 research groups with more than 700 scientists from 29 institutions closely collaborating with over 20 associated clinical groups across the country. CIBERER represents a ground‐breaking initiative for optimizing resources and facilitating synergies and cooperative projects between groups and institutions in different areas and disciplines in the field of rare diseases and overall with University hospitals across the country. The integrated research groups are clustered in seven research programs, encompassing major areas of national expertise (Table 1). Currently, the strategic objectives of CIBERER are closely aligned with the European Framework Programs of Horizon 2020 in the following manners: (1) applying next generation sequencing tools to the diagnosis and novel gene identification for monogenic diseases, while generating new tools for improving WES and WGS (Ayuso et al. 2013; Tort et al. 2013; de Castro‐Miro et al. 2014; Almoguera et al. 2015; Caparrós‐Martín et al. 2015; Codina‐Solà et al. 2015; Asencio et al. 2016; Ibañez et al. 2016; Sevilla et al. 2016); (2) developing novel gene and cell therapies for treating genetic diseases (Río et al. 2014; Sobrevals et al. 2014; García‐Gómez et al. 2016); (3) generating patient registries of international relevance, such as E‐IMD, E‐HOD, Wolfram, McArdle, Neuromuscular Diseases, and Cushing. In this field, CIBERER is improving the registries because it is acting as an institutional umbrella for many clinical Spanish groups; (4) promoting Orphan Drug Designation, such as for MNGIE, Fanconi Anemia, pyruvate kinase deficiency, and adrenoleukodystrophy; (5) hiring promising graduates to pursue a PhD in the field of rare inherited diseases; and (6) fostering participation in international consortiums, such as IRDiRC, ORPHANET, EUCERD Joint Action, and the RD Action. Nine years after its inception, CIBERER has emerged as a key player in bringing momentum and dynamics to novel discoveries and translation into clinical solutions. A significant increase in the number and quality of publications achieved by its integrating teams in the past few years (2011–2014) serves as proof of this concept (Fig. 2). It is notable that these achievements have been performed with a modest annual budget of 4.7M € in 2015, notably downsized from the initial budget of 8M € in 2008. Underscoring the major emphasis on genomic medicine, in 2014 CIBERER developed CIBERes, or CIBERER Spanish Variant Server (CSVS), a free online tool designed to improve the selection of potentially pathogenic genetic variants observed in Spanish individuals based on a repository of Spaniards exomes. Furthermore, in 2015, CIBERER launched two novel programs: (1) SPANEX, a web‐based database including the genomic variants (basically SNParrays and exomes) of 1000 healthy aged Spanish people. This is expected to be of highest utility for the prioritization and clinical interpretation of variants resulting from WES and WGS of individuals of Spanish ancestry (Dopazo et al. 2016), as this population is underrepresented in the present publically available databases, such as 1000Genomes, ExAC, and EVS. In addition to the development of algorithms for variant prioritization, SPANEX aims to establish a reference population with well‐selected controls to be used for current and future research studies, such as pharmacogenomics, haplotype segregation, susceptibility studies, migrations, founder effects, and evolutionary genetics. This dataset will also provide a reference for other populations with important Iberian contributions, such as Latin‐American populations; and (2) EnoD, a platform of experts of different clinical and molecular specialties aiming to investigate and help in the reinterpretation of complex, unsolved cases after inconclusive WES results.

**Table 1:** Organization of research groups per area within the CIBERER consortium

Research programs	
Genetic medicine	12 research groups
Inherited metabolic medicine	12 research groups + 6 ACG
Mitochondrial and neuromuscular medicine	12 research groups
Pediatric and developmental medicine	8 research groups + 4 ACG
Sensorineural pathology	7 research groups
Endocrine medicine	4 research groups + 5 ACG
Inherited cancer, hematological and dermatological diseases	7 research groups + 5 ACG

ACG, associated clinical groups.

These initiatives are pivotal in these early times of implementation of WES as a first‐tier diagnostic tool. We expect that the favorable cost‐efficiency analysis of NGS technologies and their pivotal role in diagnosis and novel gene identification (Yang et al. 2013; Solomon et al. 2016; Stark et al. 2016) will help authorities in rational decision making when covering costs through the National Health System.

## Perspectives: Integrating Research and Clinical Genetics in Genomic Centers

Many countries have recently made the decision to “transform” reference Clinical Genetic services into large Genomic Centers with the aim not only to absorb and handle the huge number of patients’ evaluations and genetic/genomic studies but also their growing complexity. Thus, The Department of Health of the United Kingdom set up “Genomics England” in 2013 ( http://www.genomicsengland.co.uk). The aim is to deliver the 100,000 Genomes Project. Genomics England is a company owned by the Department of Health in which the Secretary of State is the only shareholder. They work with a range of partners (NHS England, Health Education England, Public Health England, and 85 NHS Trusts and hospitals across England) to deliver this project. To identify and enroll participants for the 100,000 Genomes Project they have created NHS Genomic Medicine Centers (GMCs). Thirteen GMCs have been established by NHS England. Each center includes several NHS Trusts and hospitals. GMCs recruit and consent patients. They then provide DNA samples and clinical information for analysis. These centers not only are leading the way in delivering the 100,000 Genomes Project but also have established a clinical network for Genomic Medicine in all the country; in other words, they have set up the bases for an organization of Genetics/Genomics for all patients in UK. Similarly, other countries such as Italy, France, and Germany have followed the UK and are currently planning to organize the management of genetic and rare diseases in a nation‐wide fashion.

In a similar manner, in Spain the CIBERER (through the National Plan for Rare Diseases) and many researchers have made recommendations to the Ministry of Health to facilitate the creation and promotion of Genomic Centers following this model. The preliminary draft for the implementation of 4–5 Genomic Centers for all the country is currently under evaluation. This plan should also be approved (and more importantly, executed) by the regional health systems of each AC, who are nowadays facing severe budgetary restrictions, a fact that might hinder its actual implementation nation‐wide.

## Concluding Remarks

Spain has excellent standards in public medical care comparable with those of the most advanced economies in Europe. The previous two decades have been characterized by steady improvements in technological development for research and diagnostics in clinical genomics, as a part of a continuum. Together, the development of strategies and initiatives at the regional and national levels; the approval of the Clinical Genetics specialty; the empowerment of patients, nicely exemplified by FEDER (*Federación Española de Enfermedades Raras,* www.enfermedades-raras.org); and the increasing awareness of the ethical, legal, and social aspects of genetics are helping translate the newest advances to clinical practice. The Spanish society, today better educated than ever, places a great value on human rights. In this sense the task in the field of biomedicine and biology developed by the Bioethics and Law Observatory and UNESCO Chair in Bioethics from the Barcelona University (www. bioeticayderecho.ub. edu) and the Inter‐University chair in Law and the Human Genome of Universidad de Deusto and Universidad del País Vasco ( www.catedraderechoygenomahumano.es) has fostered a rigorous and cross‐disciplinary analysis of the legal implications of the advances in molecular genetics and genomics.

One difficulty, however, is the effect that the recent economic turmoil may exert on public and universal access to the highest quality services in genetics and genomic medicine. Nevertheless, we trust in the recognition by law of the fundamental right of citizens to access the best standard of care derived from the advances driven by the novel genetic technologies and knowledge in medical genomics. Fortunately, implementing NGS technologies in clinical genetics practice serves two masters, improving diagnostic efficacy and thus patient management, while saving significant costs to the public National Health System.

## Conflict of Interest

None declared.

## Notes

1 This denomination refers to the short period of restored democracy in Spain. Whereas it is clear that the origin was after Franco's death in November 1975, the end of this period is not clear. Some historians place it at the Spanish Constitution in 1978 and others at the national socialist victory (PSOE) in October 1982.

2 This name refers to the Borbon Restoration that began in 1874 and ended with the proclamation of the Second Spanish Republic in 1931.
